# Supplementary material for: Cell-type specific innervation of cortical pyramidal cells at their apical dendrites
Source: eLife. 2020 Feb 28;9:e46876. doi: 10.7554/eLife.46876 (PMC7297530; doi:10.7554/eLife.46876)
Supplement: Supplementary file 1. [file elife-46876-supp1.docx]

Supplementary File 1: Supplementary Tables

Cell-type specific innervation of cortical pyramidal cells at their apical dendrites

Ali Karimi*, Jan Odenthal*, Florian Drawitsch, Kevin M. Boergens, Moritz Helmstaedter

**Table 1**

|  | **S1** | **V2*** | **PPC*** | **ACC** | **LPtA** | **PPC-2** |
| --- | --- | --- | --- | --- | --- | --- |
| **Mouse age (postnatal days)** | 28 | 56 | 56 | 56 | 4­­­­­6 | 57 |
| **Sample location relative to bregma in mm (AP,ML)** | (-1.2, -3.9) | (-2.6,1.7) | (-2,-1.7) | (0.8,-0.15) | (-2.1, -1.3) | (-2, -1.5) |
| **Sample section thickness (µm)** | 1000 | 600 | 600 | 600 | 1000 | 600 |
| **SEM type (FEI, USA)** | Magellan | Quanta | Quanta | Quanta | Verios | Magellan |
| **Electron landing energy (KeV)** | 2.8 | 2.8 | 2.8 | 2.8 | 2.8 | 2.8 |
| **Beam current (nA)** | 3.2 | 0.2 | 0.2 | 0.11-0.2 | 0.8 | 1.6 |
| **Beam dwell time per image pixel (µs)** | 0.1 | 2.1 - 2.8 | 2.3 | 2.1 - 2.8 | 0.5 | 0.2 |
| **Tile configuration in plane (x, y)** | 3 x 3 | 1 x 2 | 1 x 2 | 1 x 3 | 4 x 5 | 5x5, 5x10 |
| **Overlap between tiles (x, y)** | (21%, 11%) | (N/A, 8%) | (N/A, 6%) | (N/A, 4.5%) | (8%, 12%) | (6%, 7%) |
| **Single tile resolution (pixels)** | 3072x2048 | 6144x4096 | 6144x4096 | 6144x4096 | 3072x2048 | 4096x3536 |
| **voxel size (nm^3^)** | 11.24x11.24x28 | 12x12x30 | 12x12x30 | 12x12x30 | 11.24x11.24x30 | 11.24x11.24x30 |
| **Final high-resolution volume (µm^3^)** | 66x89x202 | 72x91x153 | 72x93x141 | 70x141x98 | 130x110x85 | 200x(185-370)x200 |
| **Dataset distance to pial surface (µm)** | 125 | 215 | 170 | 110 | 20 | 10 |
| **Low-resolution EM volume existence**** |  |  |  |  | X | X |
| **Staining approach** | Manual | AMW assistance | AMW assistance | AMW assistance | AMW assistance | Manual |
| **Staining protocol** | Conventio-nal | Modified Hua | Modified Hua | Modified Hua | Modified Hua | Modified Hua |

* From opposing hemispheres of same animal

** Used for annotation of apical dendrite’s trunk and identification of the cell body of origin

**Experimental parameters.**

This table summarizes experimental parameters used for sample preparation and volumetric electron microscopy in 6 datasets from 5 cortical regions.

**Table 2**

|  | **V2** | **PPC** | **ACC** | **PPC-2** |
| --- | --- | --- | --- | --- |
| **50% Ethanol** | 30 min @ 4°C | 30 min @ 4°C | 30 min @ 4°C | 30 min @ RT (cooled) |
| **75% Ethanol** | 45 min @ 4°C | 45 min @ 4°C | 45 min @ 4°C | 30 min @ RT (cooled) |
| **100% Ethanol** | 45 min @  RT (room temperature) | 45 min @ RT | 45 min @ RT | 2 times, 30 min @ RT |
| **Pure acetone** | 3 times , 45 min each @ RT | 3 times , 45 min each @ RT | 3 times , 45 min each @ RT | 4 times, 20 min each @ RT |
| **50% Spurr’s resin in acetone** | 3 hr @ RT (no rotation, closed tube). Next, open tube for 90 min initial rotation + overnight @ RT | 3 hr @ RT (no rotation, closed tube). Next, open tube for 90 min initial rotation + overnight @ RT | 3 hr @ RT (no rotation, closed tube). Next, open tube for 90 min initial rotation + overnight @ RT | 4 hr @ RT, closed tube cap, slow rotation |
| **75% Spurr’s resin** | N/A | N/A | N/A | Overnight @ RT. Slow rotation, closed caps. |
| **100% Spurr’s resin** | 6 hr @ RT | 6 hr @ RT | 6 hr @ RT | 2 times, 3 hr @ RT each. No rotation |

**Dehydration and embedding times and temperatures**.

Time and duration of each dehydration and embedding step for samples from 3 cortical region (n=4).

**Table 3**

|  | **S1** | **V2** | **PPC** | **ACC** | **LPtA** | **PPC-2** |
| --- | --- | --- | --- | --- | --- | --- |
| **Main bifurcation input mapping (Fig. 1,5)** | X | X | X | X |  | X |
| **Reconstruction of all apical dendrites (Fig. 1b)** |  |  |  | X |  |  |
| **Synapse size estimation (Fig. 2d)** | X | X | X | X |  |  |
| **Spine innervation fraction (Fig. 2a-c)** |  | X | X | X | X |  |
| **Fraction of double-innervated spines (Fig. 2e)** | X | X | X | X |  |  |
| **Fractional innervation of inhibitory axons (Fig. 3,4)** | X | X | X | X |  |  |
| **More detailed cell-type comparison (Fig. 5,7)** | X (only L2) | X(only L2) | X(only L2) | X(only L2) | X | X |
| **Path distance to soma dependency (Fig. 6)** | X (only L2) | X(only L2) | X(only L2) | X(only L2) | X | X |
| **Profile of inhibitory fraction along upper cortex (Fig.7, Fig. Suppl. 1)** | X | X | X | X | X | X |

**Overview of analyses carried out in the 6 datasets.**

**Table 4**

| **Dataset layer origin** | **L1** | | | **L2** | |
| --- | --- | --- | --- | --- | --- |
| **Seed structure** | Shaft | Spine | Shaft | | Spine |
| **Layer 2** | 14,29% (n = 14) | 100% (n = 5) | 0% (n = 92) | | 97,1% (n = 35) |
| **Layer 3** | 3,7% (n = 27) | 100% (n = 16) | N/A | | N/A |
| **Layer 5tt** | 22,5% (n = 40) | 93,3% (n = 30) | N/A | | N/A |
| **Deep (Layer 3/5)** | N/A | N/A | 1,1% (n = 91) | | 98,4% (n = 61) |
| **Layer 5st** | 60% (n=10) | N/A | 44,44% (n = 9) | | N/A |

**Fraction of spine-preferring input on spine and shaft of ADs.**

The fraction of spine-preferring (putative excitatory) synapse onto shaft and spine of apical dendrites for L2-5 pyramidal cell types in layers 1 and 2. N refers to number of axons.

**Table 5**

|  | **Somatic depth relative to pia (µm)** | |
| --- | --- | --- |
| **Dataset name** | PPC-2 | LPtA |
| **Layer 2** | 208 - 250 | 268 - 295 |
| **Layer 2MN (n=2)** | 166, 172 | N/A |
| **Layer 3** | 305 - 376 | 327 - 442 |
| **Layer 5tt** | 540 - 599 | 621 - 658 |
| **Layer 5st** | 529 - 640 | N/A |

**Somatic depth of pyramidal neurons residing in layers 2-5 (n = 40, 11 for PPC-2 and LPtA, respectively).**
